# Immunoinflammatory Profile in Patients with Episodic and Continuous Paranoid Schizophrenia

**DOI:** 10.17816/CP66

**Published:** 2021-03-20

**Authors:** Irina K. Malashenkova, Sergey A. Krynskiy, Daniil P. Ogurtsov, Nikita A. Hailov, Natalia V. Zakharova, Lidia V. Bravve, Maria A. Kaydan, Ekaterina I. Chekulaeva, Denis S. Andreyuk, Vadim L. Ushakov, Nikolay A. Didkovsky, Georgy P. Kostyuk

**Affiliations:** Laboratory of Molecular Immunology and Virology at the National Research Center, Kurchatov Institute; Federal Research and Clinical Centre of Physical-Chemical Medicine, Federal Medical Biological Agency of Russia; Mental-health Clinic No. 1, named after N.A. Alekseev

**Keywords:** adaptive immunity, cytokines, inflammation, innate immunity, schizophrenia, приобретенный иммунитет, цитокины, воспаление, врожденный иммунитет, шизофрения

## Abstract

**Introduction.:**

Associations of disturbances in innate and adaptive immunity during the clinical course of schizophrenia have been found in a number of studies. Yet, the relationship of immune parameters and systemic inflammation in relation to the clinical course of the disease and its prognosis, remains poorly understood, which highlights an interesting topic for further research. The goal of this study was to research the immuno-inflammatory changes in patients with clinical continuous and episodic paranoid schizophrenia, to assess the pathogenetic significance of these changes.

**Methods.:**

Thirty-six patients with paranoid schizophrenia, of which 20 had episodic symptoms and 16 had continuous symptoms, consented to participate in the study, together with 30 healthy volunteers. In the study we assessed the parameters of innate immune response (serum levels of key pro-inflammatory and anti-inflammatory cytokines, C-reactive protein) and the adaptive immune response, including humoral-mediated immunity (serum immunoglobulins IgA, IgM, IgG, circulating immune complexes), as well as the cell link of adaptive immunity (key lymphocyte subpopulations). Positive and negative symptoms were assessed with the positive and negative symptoms scale; frontal dysfunction was assessed by Frontal Assessment Battery (FAB).

**Results.:**

Both patient groups had higher than normal levels of C-reactive protein and IL-8. There was a significant elevation of circulating immune complexes among patients with continuous symptoms of schizophrenia, compared to patients with episodic symptoms and healthy controls. Levels of CD45+CD3+ lymphocytes (T-cells) differed between clinical groups, with higher values identified among patients with episodic symptoms and lower values among those with continuous symptoms. In addition, patients with episodic symptoms had significantly increased levels of CD45+CD3+CD4+CD25+CD127- regulatory T-cells. Finally, the level of CD45+CD3-CD19+ B-cells was significantly higher among patients with continuous symptoms vs. patients with episodic symptoms and the control groups. Markers of activation of humoral immunity were associated with the severity of frontal disorders in these patients.

**Discussion.:**

Comprehensive data on the serum level of cytokines and the parameters of adaptive immunity among individuals with continuous schizophrenia, by comparison with patients with episodic schizophrenia, are practically absent in the literature. We have shown that among those with continuous schizophrenia, there are signs of systemic inflammation and chronic activation of the adaptive humoral immune response, while among patients with episodic symptoms of the disease, there are signs of systemic inflammation and certain activation of cell-mediated immunity, without significant changes in the humoral link of adaptive immunity.

**Conclusion.:**

More studies are needed, but the data obtained in this study are important for subsequent clinical studies of new treatment methods, based on various immunophenotypes of schizophrenia.

## INTRODUCTION

Schizophrenia is a polymorphic mental illness, characterized by disorders of thinking, perception, impairments of memory, attention and executive functions. The prevalence of schizophrenia in Russia is around 1%, with most cases among young adults (peak incidences occur between the ages of 15 and 35). The socio-economic burden, associated with schizophrenia, is determined by a high percentage of disability and by high costs of treatment and maintenance [1].

Signs of neuroinflammation, including chronic excessive activation of microglia and astrocytes, are found in schizophrenia [2]. According to the “mild encephalitis” hypothesis, low intensity neuroinflammation is a key pathogenetic mechanism in some patients [3]. It is suggested that neuroinflammation in schizophrenia can be attributed to infectious, autoimmune and traumatic factors, but its exact causes remain unknown [2,4].

**Figure 1:**
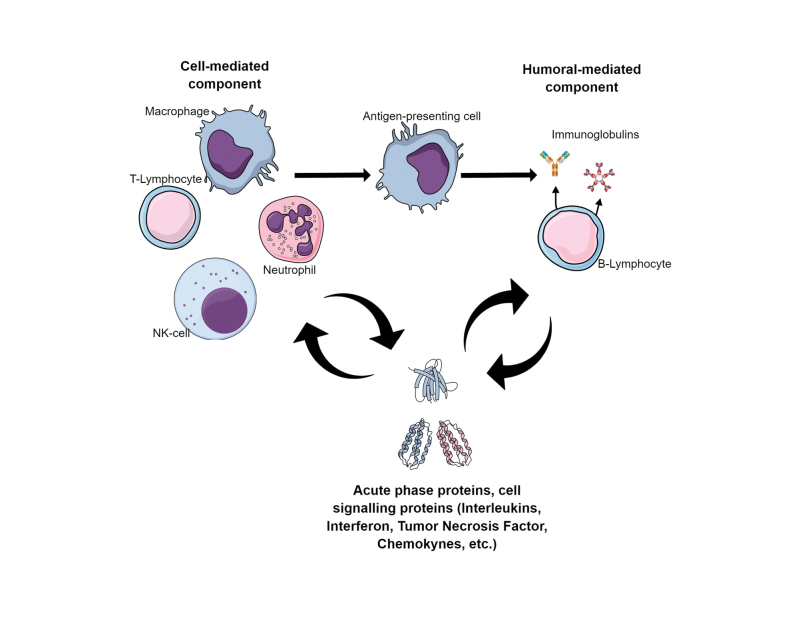
Figure 1. Key mechanisms of the immune response

A series of evidence is being discussed relating to the role of systemic inflammation in the pathogenesis of schizophrenia [3,5-10]. There is a hypothesis that one of the leading components of the pathogenesis of this disease is immune dysfunction, associated with an increased risk of infections and autoimmune disorders (Figure 1). It has been shown that patients with schizophrenia have higher rates of exposure to pathogens such as toxoplasma gondii, cytomegalovirus and the human herpesvirus type 6. Patients with schizophrenia have an elevated risk of death from infectious diseases compared to controls, and a history of autoimmune disease has been associated with a 45% increase in the risk of developing schizophrenia [4,11-14].

The role of immune disorders in the pathogenesis of schizophrenia is supported by epidemiological and molecular biological data. Thus, according to several studies, including meta-analyses, there are signs of increased activation of systemic inflammation among patients with schizophrenia, including elevated levels of the proinflammatory cytokines, including interleukin-1β (IL-1β), IL-8 and IL-6 in both blood serum and cerebrospinal fluid [5-9]. Schizophrenia is also characterized by an increase in the serum level of neutrophil activation markers that contribute to systemic inflammation (leukocyte elastase, a1-proteinase inhibitor), and the levels of these markers correlate with the activity of the disease [10] In patients with nonpsychotic mental disorders, changes in the level of neutrophil activation markers are much less common [15]. In addition, the serum concentration of the chemokine, CCL2, which is involved in the migration of monocytes, memory T-cells and dendritic cells to the sites of inflammation, is increased in patients with schizophrenia [16].

Certain studies have found associations of immune disturbances with clinical schizophrenia [5,17-19] In the case of chronic schizophrenia, the proinflammatory cytokine profile was found to be different by comparison with acute schizophrenia, with increased levels of IL-1β and IL-6, however, no changes were evident with regard to other proinflammatory cytokines [5] Increased CCL2 in schizophrenia is associated with treatment resistance, and high levels of C-reactive protein (CRP), an inflammatory marker, are associated with a more severe form of psychosis and a subsequent decrease in cognitive functions [17-19] According to another study, the fluency of speech of patients with schizophrenia and schizoaffective disorder, that have elevated expression of the cytokines IL-1β, IL-2, IL-6, IL-8 and IL-18 in the blood, is significantly more impaired [20]. In addition, a 17% decrease in the volume of the left Broca's area was observed among patients with a high level of cytokines [20].

Signs of neuroinflammation, most pronounced in the areas of the cortex that are most affected by the disease, are also found in studies of the post-mortal brain of patients with schizophrenia [21,22]. In the prefrontal cortex of these patients, the mRNA level of the Interferon-Induced Transmembrane Protein (IFITM) is increased. One of the effects of the IFITM protein is the activation of the transcription factor NF-κB, which is a key factor in inflammatory cascades [21]. The expression of the proinflammatory cytokines, IL-1β, IL-6 and TNFα is also increased in the prefrontal cortex of patients [22].

Among patients with schizophrenia, a number of changes in adaptive immunity are also present [23-25]. According to a meta-analysis of 16 works, the level of CD4 + T-helper cells, CD16 + CD56 + NK cells, naive B-cells and CXCR5 + memory T-cells, was increased among those with schizophrenia [24,25]. However, according to other data, the level of T-helper cells, as well as the ratio of CD4 + / CD8 + in schizophrenia are reduced, and the patients have impairments of T-cell activation [23,26]. Perhaps these contradictions are associated with different stages and forms of the disease of the examined patients.

There are data that suggest that the neutrophil/lymphocyte ratio, platelet/lymphocyte ratio and monocyte/lymphocyte ratio are higher during the relapse periods of schizophrenia, compared to the remission periods [27]. According to another study, inflammatory activation in schizophrenia, including excessive lymphocyte and monocyte activation, is currently independent of cardiometabolic risk factors [28]. Currently, studies of the effectiveness of anti-inflammatory drugs and immunomodulating agents in the treatment of schizophrenia are also of great interest [29]. Thus, research data indicate that there are numerous signs of systemic inflammatory response and dysregulation of the adaptive immunity in schizophrenia. However, in terms of the perplexity of the immune response (Figure 1), there is a lack of research into multiple immune parameters in schizophrenia [30] and their relationships in terms of the clinical course of schizophrenia and its outcomes; this is a promising aspect of the investigation shown in Figure 1.

Both the innate and the adaptive immune system involve a cell-mediated (immune cells) and humoral-mediated (immunoglobulins and complement) response. The activation of innate immune cells causes inflammation and stimulates the presentation of antigens to adaptive immune cells (T-lymphocytes and B-lymphocytes). When facing an antigen, the T-lymphocytes mediate cytotoxicity against the infected or altered cells and activate B-lymphocytes to proliferate and produce immunoglobulins. The activated immune cells are orchestrated by the variety of cytokines and produce acute phase proteins.

The goal of this research was to study the immuno-inflammatory changes among patients with various clinical symptoms of paranoid schizophrenia (episodic and continuous) compared with healthy controls. This was conducted in order to assess the associations of immunological changes with clinical symptoms and to provide a сoncurrent measurement of multiple immune parameters in both episodic and continuous schizophrenia.

## MATERIAL AND METHODS

The study included 36 patients with paranoid schizophrenia (see Table 1) and 30 healthy volunteers with no mental disorders (13 men, 17 women), comparable in gender. The mean (± 95% confidence interval) age of the volunteers was 27.1±1.6 years, ranging from 23 to 33 years. The duration of maintenance antipsychotic therapy among patients, ranged from six months to two years; the duration of hospitalization before the assessment was three to four weeks. The compliance of the participants mental state at the time of the examination with the criteria for schizophrenia, according to ICD-10, constituted the inclusion criteria; informed written consent was also required to participate in the study. Patients with recurrent symptoms of schizophrenia (n=13) / first psychotic episode (n=7) (Group 1) and patients with continuous symptoms of schizophrenia (n=16) (Group 2) were included in the study. Continuous schizophrenia was defined clinically, according to DSM-5 criteria, by persistent symptoms fulfilling the diagnostic symptom criteria of the disorder that remained throughout the duration of the illness. Subthreshold symptom periods were very brief in relation to the overall symptoms and it was impossible to define distinct episodes. Among patients with episodic symptoms, the defect was stable and there were distinct psychotic episodes, with symptomatic remissions lasting more than six months. The exclusion criteria were severe somatic diseases, pregnancy, acute or exacerbated, chronic, infectious and inflammatory diseases during the two months preceding the examination or signs of drug or alcohol abuse.

**Table 1:** Table 1. Socio-demographic characteristics of patients included in the study p - values for t-test are provided for differences between the two groups. 1 Results are presented as means (± standard deviation), statistics – as t and p (t-test) 2 Results are presented as n (%), statistics – as χ2 and p

Value/Groups	Continuous schizophrenia(n=16)	Episodic schizophrenia(n=20)	Control (n=30)	Statistics
Age on the date of assessment 1	28.6 ± 6.8	27.6 ± 7.2	27.1 ± 1.6	t=-0.424; p=0.674
Gender distribution				
Male 2	14 (88)	9 (45)	13 (43)	χ2=9.264; p=0.01
Female 2	2 (13)	11 (55)	17 (57)	
Family				
Married 2	2 (13)	3 (15)	13 (43)	χ2=8.277; p= 0.082
Divorced 2	1 (6)	1 (5)	-	
Never married 2	13 (81)	16 (80)	17 (57)	
Education				
Lower secondary 2	1 (6)	3 (15)	-	χ2=29.597; p<0.001
Secondary 2	2 (13)	3 (15)	-	
Specialized secondary 2	4 (25)	1 (5)	-	
Incomplete higher 2	6 (38)	4 (20)	4 (13)	
Higher 2	3 (19)	9 (45)	26 (87)	
Duration of education in years 1	13.9 ± 1.5	13.6 ± 2.5	16.6 ± 1.0	t=-0.422; p=0.675
Labor status				
Student 2	2 (13)	5 (25)	9 (30)	χ2=29.545; p<0.001
Employed 2	2 (13)	7 (35)	19 (63)	
Unemployed, pre-retirement 2	5 (31)	7 (35)	-	
Disabled, absolute (%)	7 (44)	1 (5)	2 (7)	
Age of prodromal symptoms onset 1	15.3 ± 3.8	20 ± 5.9	-	t=2.758; p=0.009
Age of manifest 1	19.2 ± 5.2	25 ± 6.3	-	t=2.961; p=0.006
Age of first apply for medical help 1	19.2 ± 4	25.7 ± 7	-	t=3.302; p=0.002
Age of first hospitalisation 1	19.3 ± 4.1	25.7 ± 6.9	-	t=3.271; p=0.003
Duration of illness from the prodromal symptoms 1	13.3 ± 6.2	7.7 ± 4	-	t=-3.281; p=0.002
Duration of illness from the manifest 1	9.4 ± 6.3	2.7 ± 3.2	-	t=-4.144; p<0.001

The PANSS, BFCRS, Simpson-Angus Scale (SAS), Symptom Severity Scale of the DSM5 for schizophrenia (SS-DSM5) and the FAB scale were used in clinical assessments [31].

We measured the parameters of humoral-mediated immunity (levels of Circulating Immune Complexes (CIC), immunoglobulins IgA, IgM, IgG), levels of signalling (IL-4, IL-6, IL-8, IL-10, IFNγ, TNFα) and acute phase (CRP) proteins. The parameters of the cell link of adaptive immunity were evaluated by multicolour flow cytometry, using monoclonal antibodies for the phenotyping of the differentiation antigens, CD3, CD4, CD8, CD16, CD19, CD25, CD45, CD56 and CD127 (Becton Dickinson, USA). To determine the concentration of proinflammatory cytokines (IL-4, IL-6, IL-8, IFNγ, TNFα), the pro-inflammatory acute phase protein CRP, the anti-inflammatory cytokine, IL-10, the markers of the humoral link of adaptive immunity CIC, the immunoglobulins (IgA, IgM, IgG) in blood serum by enzyme-linked immunosorbent assay (ELISA) and the commercial kits for ELISA by "Cytokine", "Vector Best" and "Hema" (Russia) were used.

For statistical processing, Excel software (Microsoft, 2010) and STATISTICA 10 (StatSoft, 2010) were used. The Shapiro-Wilk test was used to assess the normality of distribution. The results of immunological tests were presented as medians with an interquartile range. The Kruskell-Wallis test was used to assess the significance of differences between groups, with post-hoc, pair-wise comparisons conducted using the Mann-Whitney test. The results of the clinical assessment (age, duration of education and illness, results of clinical scales) demonstrated a normal distribution and were presented as means +- standard deviations. The Student’s t-test was used to assess the significance of differences in the results of the clinical assessment between groups. The frequencies distribution between groups was tested with the Chi-square test with continuity correction. The differences between groups were considered statistically significant at two-sided p <0.05. To estimate the correlations between variables, the Pearson correlation coefficient was used.

## RESULTS

Table 1 shows the socio-demographic characteristics of the participants. The patients generally had fewer opportunities to progress to higher education or to be employed.

The clinical characteristics of patients are presented in Table 2. As can be seen, in the continuous course of schizophrenia compared with the episodic course, the patients exhibited a significantly greater severity of negative symptoms and cognitive disorders.

**Table 2:** Table 2. Clinical indicators of patients included in the study

Scale	Episodic schizophrenia (n=20)	Continuous schizophrenia (n=16)	t-test
PANSS total	89.9 [81.1, 98.7]	112.6 [105.5, 119.7]	t=4.075; p<0.001
PANSS P	24.1 [20.2, 28]	25.6 [21.4, 29.8]	t=0.550; p=0.586
PANSS N	20.6 [17.1, 24.1]	32.9 [30.3, 35.5]	t=5.675; p<0.001
PANSS G	45.2 [41.4, 49]	53.8 [50.2, 57.4]	t=3.397; p=0.002
BFCRS	6 [2.6, 9.4]	8.3 [3.3, 13.3]	t=0.830; p=0.413
NSA-4	13.7 [11.6, 15.8]	24.2 [21.7, 26.7]	t=6.837; p<0.001
Simpson-Angus Scale (SAS)	1.6 [0.7, 2.5]	2.4 [1.4, 3.4]	t=1.254; p=0.219
Symptom severity scale of the DSM5 for schizophrenia (SS-DSM5)	12.3 [11.1, 13.5]	15.4 [14.1, 16.7]	t=3.682; p=0.001
Frontal assessment battery (FAB)	15.1 [14.3, 15.9]	13.2 [12.1, 14.3]	t=-3.022; p=0.005

Table 3 summarizes the data on the immune parameters of patients with episodic and continuous symptoms, and those of the healthy control group. The assessment of the markers of systemic inflammation and adaptive immunity has shown that the levels in patients with schizophrenia differed significantly from those in the control group; these levels were characterized by pronounced heterogeneity and were affected by the course of the disease (Table 3).

**Table 3:** Table 3. Immunological parameters and systemic inflammation markers among patients with episodic schizophrenia (n=20), continuous schizophrenia (n=16) and among the controls (n=30) The results are presented as Median (25 quartile; 75 quartile). * - differences with controls, p<0.05. ** - differences between groups, p<0.05.

Parameters		Episodic schizophrenia	Continuous schizophrenia	Controls
Humoral component parameters	IgA, [g/l]	2.97 (2.31; 3.35)	3.09 (2.17; 3.23)	2.79 (2.58; 3.09)
	IgM, [g/l]	1.43 (0.93; 2.09)	1.05 (0.73; 1.46)	0.9435 (0.71; 1.3)
	IgG, [g/l]	13.69 (9.73; 15.19)	13.62 (11.07; 15.81)	12.74 (10.15; 14.44)
	CIC, [units]	73 (58; 104)**	103 (84; 192)*,**	76 (54; 95.5)
Acute phase proteins, signalling proteins (cytokines)	CRP, [mg/l]	5.48 (0.81; 20.33)*	3.3 (1.93; 8.4)*	1.08 (0.38; 2.53)
	IL-4, [pg/ml]	2.33 (1.72; 4.61)	3.5 (1.06; 5.13)	4.19 (1.39; 8.31)
	IL-10, [pg/ml]	5.03 (2.62; 6.76)	5.91(3.03; 8.28)	4.28 (1.39; 8.31)
	IFNγ, [pg/ml]	44.69 (26.88; 138.78)	44.69 (19.38; 83.33)	28.5335 (20.31; 44.32)
	IL-8, [pg/ml]	34.54 (11.19; 214.76)*	40.16 (12.61; 94.15)*	11.81 (7.31; 23.59)
	TNFα, [pg/ml]	2.09 (1.75; 3.08)	1.62 (1.30; 2.23)	1.69 (1.40; 2.84)
	IL-6, [pg/ml]	6.27 (4.02; 67.20)	5.39 (2.55; 10.85)	4.262 (2.42; 8.27)
Cell-mediated component parameters	CD45+CD3+, [%]	77.60 (74.60; 79.50)**	71.05 (68.40; 74.20)**	75.95 (71.85;78.20)
	CD45+CD3+CD4+, [%]	44.00 (39.20; 47.70)	39.95 (38.10; 44.00)	43.65 (36.60; 48.40)
	CD45+CD3+CD8+, [%]	25.8 21.40; 31.20)	23.85 (22.00; 28.80)	26.3 (23.8; 27.00)
	CD45+CD3+CD4+CD25+CD127-, [%]	3.3(2.65; 3.45)	1.85 (1.40; 3.05)	2.45 (2.15; 3.30)
	CD45+CD3+CD4+CD25+CD127- % from CD45+CD3+CD4+ lymphocytes, [%]	6.85 (6.05; 7.50)*	4.90 (3.6; 6.9)	4.75 (4.60; 5.55)
	CD45+CD3-CD16/56+, [%]	10.60 (7.10; 13.40)	11.90 (9.60; 16.50)	11.90 (8.30; 16.50)
	CD45+CD3+CD16/56+, [%]	3.40 (2.40; 6.40)	3.90 (2.60; 8.00)	4.9 (1.30; 7.50)
	CD45+CD3-CD19+, [%]	10.40 (8.00; 13.00)**	14.00 (11.00; 18.30)*,**	11.40 (9.70; 12.20)

### Humoral component of immune system

There was a significant elevation of CIC in patients with continuous symptoms, by comparison with patients with episodic symptoms and healthy controls, whereas the levels of immunoglobulins did not differ.

### Cell-mediated immunity

The levels of CD45+CD3+ lymphocytes (T-cells) differed between clinical groups, with higher values among those with episodic symptoms and lower values among those with continuous symptoms. Despite the fact that their levels in control group had intermediate values, there were no significant differences among the clinical groups of the controls. In addition, patients with episodic symptoms had significantly increased levels of CD45+CD3+CD4+CD25+CD127- regulatory T-cells. Finally, the level of CD45+CD3-CD19+ B-cells were significantly higher among the patients with continuous symptoms vs. patients with episodic symptoms and among the control groups.

### Levels of cytokines and acute phase proteins

Both patient groups had higher than normal levels of CRP and IL-8, while the levels of other cytokines did not differ compared to the control group.

### Correlations with clinical scores

Among patients with episodic symptoms, a moderate, negative correlation of the level of the anti-inflammatory cytokine, IL-10, with NSA-4 scale results was revealed (r = -0.55; p <0.01).

Among patients with continuous symptoms, the level of CD45+CD3-CD19+ B cells and the level of the CIC showed a moderate, negative correlation with the FAB scale results (r = -0.60; p <0.01), thus, a high level of activation of humoral immunity in this subgroup of patients, was associated with more pronounced cognitive impairment.

Therefore, the patients with continuous schizophrenia had an excessive activation of humoral immunity, that was associated with the severity of cognitive disorders and a moderate activation of systemic inflammation.

## DISCUSSION

**Figure 2:**
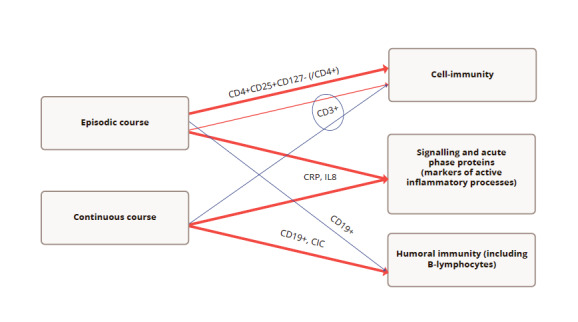
Figure 2. Key results of the study. Note: red – increase vs. control, dark blue – decrease vs. control. Thick line – statistically significant difference vs. control; thin – non-significant difference vs. control; circle – difference between clinical groups. CRP – marker of active inflammation; IL8 – proinflammatory chemokine; CD19+ - B-lymphocytes; CIC – CD3+ T-cells; CD4+CD25+CD127- (/CD4+) - regulatory T-cells (% from CD45+CD3+CD4+ lymphocytes)

In this study we found multiple signs of immunological disturbances that differ, depending on the clinical course of schizophrenia. The main findings are summarized in Figure 2 below. While the levels of CRP and IL8 were increased in schizophrenia, irrespective of clinical groups, the patients with episodic symptoms demonstrated a significant increase of CD45+CD4+CD25+CD127- (/CD4+) cells, and patients with continuous symptoms had increased levels of CD45+CD3-CD19, B-cells and CIC vs. the control group. Interestingly, the numbers of CD45+CD3+ lymphocytes (T-cells) differed significantly among the clinical groups but not among the controls, were slightly increased among patients with episodic symptoms and were slightly decreased among patients with continuous symptoms.

Therefore, in summary, patients with episodic symptoms of schizophrenia were characterized by pronounced signs of systemic inflammation and a certain activation of cell-mediated immunity, without significant changes in the humoral link immunity. Conversely, patients with continuous schizophrenia had signs of active systemic inflammation and chronic activation of the humoral immune response.

An analysis of the literature shows that the relationship of systemic inflammation and dysregulation of adaptive immunity in relation to the clinical course of schizophrenia, have not been extensively studied. There are data which demonstrate that in patients with the first episode of schizophrenia, the level of IL-1β, IL-2, IL-8, IL-4, IFNα and TNFα increased, and the content of pro-inflammatory cytokines, IL-1β, IL-8 and TNFα remained increased even after eight weeks of antipsychotic therapy with risperidone or haloperidol [32]. A limitation of this work was that it did not include patients with repeated psychotic episodes and with continuous symptoms of the disease. Another study has shown that changes in the clinical blood test (neutrophil/lymphocyte ratio, platelet/lymphocyte ratio and monocyte/lymphocyte ratio) are more prominent during the relapse periods of schizophrenia, compared to remission periods [27].

In our study there was no significant increase of TNFα among patients with episodic or continuous schizophrenia, although IL-8 was increased. Certain authors report an increase in serum TNFα among patients with schizophrenia. One possible reason for this discrepancy is that antipsychotic therapy reduces the production of certain proinflammatory cytokines in schizophrenia, including TNFα [33]. The extent of this effect has not been studied in detail and may be dependent on the duration of therapy and the drugs used.

According to in vitro studies, conducted by Ryazantseva et al. among patients with schizophrenia, there is an imbalance in the production of Th1 and Th2 cytokines by lymphocytes and signs of inhibition of the T-cell link of the immune system; these changes are most pronounced with regard to chronic symptoms of the disease [34]. The results are consistent with our data on the serum levels of these cytokines. According to a meta-analysis [5] in acute schizophrenia, the levels of IL-6, TNFα and the receptor antagonist, IL-1RA are elevated, while in chronic schizophrenia, the same meta-analysis showed an increase in the levels of IL-1β and IL-6. Acute schizophrenia was defined as hospitalization in connection with a psychotic episode or chronic schizophrenia, following an examination of patients receiving outpatient treatment. It should be noted that comprehensive data on the serum level of the main cytokines and the characteristics of adaptive immunity among those with continuous symptoms of schizophrenia, by comparison with episodic symptoms, are practically absent in the literature. We have shown for the first time that in relation to continuous schizophrenia, changes in the adaptive immune response with a predominance of activation of its humoral link are of primary importance, although moderate signs of activation of systemic inflammation, do persist in patients. In particular, an increase in the content of CIC, which are formed as a result of the binding of exogenous or endogenous antigens by immunoglobulins (antibodies), indicates an inflammatory process with activation of the humoral link of adaptive immunity. The causes of this activation remain to be studied. It is possible that the activation of autoimmune processes may take place among certain patients

According to the literature (see, for example, a review [35]), in a number of schizophrenic patients, antibodies to NMDA receptors and other central nervous system proteins are detected in the peripheral bloodstream. Based on our results, it can be assumed that patients with continuous symptoms of the disease and signs of excessive activation of humoral immunity, are a risk group for the possible presence of autoimmune diseases, and an examination may be necessary in these patients to exclude a hidden autoimmune pathology.

According to our data, with regard to the episodic course of the disease, there was a pronounced complex activation of the mechanisms of systemic inflammation. An increase in CRP is a marker of acute and chronic inflammatory processes, both infectious and endogenous. In addition, there was an increase in the level of the pro-inflammatory cytokine, IL-8, which is involved in the chemotaxis of neutrophils, monocytes and lymphocytes to the site of the inflammatory reaction. There was also an increase in regulatory T-cells, which, taking into account the increased level of a number of markers of systemic inflammation, could indicate compensatory activation of immunoregulatory mechanisms. Further studies are required to determine the causes and clinical significance of these changes. At the same time, the activation of immunoregulatory mechanisms in certain patients may have a protective role and also requires further study.

A correlation analysis showed that among patients with continuous symptoms of schizophrenia, the severity of cognitive impairment was associated with a higher level of CIC and B-cells, and among those with episodic symptoms, the level of the anti-inflammatory cytokine, IL-10, was associated with clinical indicators; it negatively correlated with the severity of negative symptoms in patients. These novel data confirm the possible clinical and pathogenetic significance of the detected immune changes in various types of schizophrenia, and indicate the prospect of studying the effectiveness of various approaches to immunomodulating therapy, within the framework of the comprehensive rehabilitation of patients with schizophrenia, depending on the clinical course and immune disorders.

According to contemporary theoretical concepts, the heterogeneity of immunological changes in schizophrenia may reflect the presence of several immunophenotypes of the disease, with various pathogenesis features. Thus, it is assumed that there is an immunophenotype with a predominance of activation of systemic inflammation, the main marker of which may be an increased level of IL-6, and an immunophenotype with excessive activation of the humoral link of adaptive immunity, accompanied by an increase in the level of autoantibodies to NMDA receptors [28]. Schizophrenia immunophenotypes may have prognostic features and may require different approaches to therapy, but at the same time, an analysis of the available literature shows that their relationship with the clinical characteristics of the disease is not well understood, limiting the possibility of clinical data translation. Our results show that patients with an episodic course of schizophrenia differ in prevailing immune changes from patients with a continuous course. The data obtained may be important for the development of personalized approaches to immunotherapy in various variants of the clinical course of schizophrenia.

Discussing the limitations of this work, it should be noted that it is of interest to study immunological changes in other variants of the course of schizophrenia, that have not been studied in the framework of the present study. It should also be noted that the patients were enrolled into the study after three to four weeks of hospital treatment, and the immune parameters could be affected by antipsychotic treatment. In addition, it would be important to replicate the results in larger studies, applying the correction for multiple testing.

## CONCLUSION

An analysis of available literature shows that studies providing a comprehensive assessment of the markers of the cell and the humoral link of adaptive immunity, in combination with the parameters of systemic inflammation in schizophrenia, are still lacking. Virtually no study has been undertaken regarding the level of immunoinflammatory markers, depending on the clinical dynamics of schizophrenia: in most studies, patients are considered within the general sample or a division into acute and chronic schizophrenia is carried out. Very few works are devoted to the study of immunoinflammatory disorders in schizophrenia, examining the nature of the course and the prevailing symptoms, as well as studying the relationship between immunological parameters and neurophysiological changes in patients. This study made it possible to obtain new data on the characteristics of the cytokine profile and the nature of the main changes in the immune response depending on the course of schizophrenia. This helped determine the relationship between multiple immunological and clinical changes among patients with continuous and episodic symptoms of schizophrenia. The data obtained are important for future clinical studies of new treatment methods, based on the medical and physical methods of correction of systemic disorders, in various immunophenotypes of schizophrenia.

## Author Contributions

Irina K. Malashenkova: developing the research design, article writing, reviewing of publications on the article’s theme, revising the analysis; Sergey A. Krynskiy: experiments, statistical analysis, article writing, reviewing of publications on the article’s theme; Daniil P. Ogertsov: experiments, statistical analysis, reviewing of publications on the article’s theme, designing the figures; Nikita A. Hailov: experiments; Natalia S. Zakharova: obtaining data for analysis, collection and analysis of clinical data; Lidia V. Bravve: obtaining data for analysis, collection and analysis of clinical data; Maria A. Kaydan: obtaining data for analysis, collection and analysis of clinical data; Ekaterina I. Chekulaeva: experiments; Denis S. Andreyuk: developing and coordinating of the research design; Vadim L. Ushakov: reviewing of publications on the article’s theme; Nikolay A. Didkovsky: reviewing of publications on the article’s theme; revising the manuscript; Georgy P. Kostyuk: developing and coordinating the research design.

## Funding

This research was funded by NRC "Kurchatov Institute" (order №1361, 25.06.2019) and partially by RFFК (grant №17-29-02518).

## Conflict of Interest

The authors declare no conflicts of interest.

## Informed Consent

All patients gave written informed consent to participat in the study.
